# Tubular biomarkers in proteinuric kidney disease: histology correlation and kidney prognosis of tubular biomarkers

**DOI:** 10.1093/ckj/sfae146

**Published:** 2024-05-09

**Authors:** Javier Carbayo, Úrsula Verdalles, Francisco Díaz-Crespo, Alberto Lázaro, Marian González-Nicolás, David Arroyo, David Blanco, Mercedes García-Gámiz, Marian Goicoechea

**Affiliations:** Department of Nephrology, Hospital General Universitario Gregorio Marañón, Madrid, Spain; Department of Nephrology, Hospital General Universitario Gregorio Marañón, Madrid, Spain; Department of Pathology, Hospital General Universitario Gregorio Marañón, Madrid, Spain; Renal Pathophysiology Laboratory, Instituto Investigación Sanitaria Gregorio Marañón, Madrid, Spain; Renal Pathophysiology Laboratory, Instituto Investigación Sanitaria Gregorio Marañón, Madrid, Spain; Department of Nephrology, Hospital General Universitario Gregorio Marañón, Madrid, Spain; Renal Pathophysiology Laboratory, Instituto Investigación Sanitaria Gregorio Marañón, Madrid, Spain; Department of Biochemistry, Hospital General Universitario Gregorio Marañón, Madrid, Spain; Department of Nephrology, Hospital General Universitario Gregorio Marañón, Madrid, Spain

**Keywords:** cortical interstitial inflammation, interstitial fibrosis, MCP-1, tubular biomarkers, uDKK3, uromodulin

## Abstract

**Background:**

Proteinuria is not only a biomarker of chronic kidney disease (CKD) but also a driver of CKD progression. The aim of this study was to evaluate serum and urinary tubular biomarkers in patients with biopsied proteinuric kidney disease and to correlate them with histology and kidney outcomes.

**Methods:**

A single-center retrospective study was conducted on a cohort of 156 patients from January 2016 to December 2021. The following urinary and serum biomarkers were analyzed on the day of kidney biopsy: beta 2 microglobulin (β2-mcg), alpha 1 microglobulin (α1-mcg), neutrophil gelatinase-associated lipocalin (NGAL), urinary kidney injury molecule-1 (uKIM-1), monocyte chemoattractant protein-1 (MCP-1), urinary Dickkopf-3 (uDKK3), uromodulin (urinary uUMOD), serum kidney injury molecule-1 (sKIM-1) and serum uromodulin (sUMOD). A composite outcome of kidney progression or death was recorded during a median follow-up period of 26 months.

**Results:**

Multivariate regression analysis identified sUMOD (β–0.357, *P* < .001) and uDKK3 (β 0.483, *P* < .001) as independent predictors of interstitial fibrosis, adjusted for age, estimated glomerular filtration rate (eGFR) and log proteinuria. Elevated levels of MCP-1 [odds ratio 15.61, 95% confidence interval (CI) 3.52–69.20] were associated with a higher risk of cortical interstitial inflammation >10% adjusted for eGFR, log proteinuria and microhematuria. Upper tertiles of uDKK3 were associated with greater eGFR decline during follow-up. Although not a predictor of the composite outcome, doubling of uDKK3 was a predictor of kidney events (hazard ratio 2.26, 95% CI 1.04–4.94) after adjustment for interstitial fibrosis, eGFR and proteinuria.

**Conclusions:**

Tubular markers may have prognostic value in proteinuric kidney disease, correlating with specific histologic parameters and identifying cases at higher risk of CKD progression.

KEY LEARNING POINTS **What was known:**

- Proteinuria is not only a biomarker of chronic kidney disease (CKD) but also a driver of CKD progression, exerting a toxic effect on tubular cells and promoting tubular atrophy and interstitial fibrosis.
- Tubular biomarkers have been evaluated in acute kidney injury and in CKD populations, but have not been specifically studied in patients with glomerular diseases and high-grade proteinuria, often lacking histologic correlation, and limiting the evaluation to a single tubular marker.
- We aimed to evaluate seven different serum and urinary tubular biomarkers in patients with biopsied proteinuric kidney disease and to correlate them with histology and kidney outcomes.

**This study adds:**

- We found a correlation between certain tubular biomarkers and median interstitial fibrosis in proteinuric patients. Urinary Dickkopf-3 (uDKK3) emerged as a strong and independent predictor of interstitial fibrosis after adjustment for age, estimated glomerular filtration rate (eGFR) and proteinuria.
- Monocyte chemoattractant protein-1 (MCP-1) correlated with cortical interstitial inflammation, independent of age, eGFR, proteinuria and microhematuria.
- uDKK3 levels at the time of kidney biopsy were associated with eGFR decline during follow-up and was the only biomarker associated with kidney events but not the composite outcome.

**Potential impact:**

- The use of specific tubular biomarkers may have prognostic value in proteinuric kidney disease by correlating with specific histologic parameters and identifying potential cases at higher risk of CKD progression in this population.
- The evaluation of tubular biomarkers could provide a comprehensive and holistic approach to patients with proteinuric kidney disease.

## INTRODUCTION

Proteinuria is considered the primary biomarker of chronic kidney disease (CKD) progression and is associated with an increased risk of cardiovascular events and adverse outcomes [1, 2]. However, proteinuria is not only an expression of kidney damage but also a driver of CKD progression [3]. Excessive filtration of plasma proteins across the glomerular filtration barrier exerts a toxic effect on tubular cells, promoting the activation of multiple pathways that ultimately lead to tubular atrophy and fibrosis and end-stage kidney disease (ESKD) [4, 5]. Tubular epithelial cells are highly metabolically active and particularly susceptible to damage caused by hypoxia, toxins, inflammatory insults or proteinuria [6–11].

Glomerular and proteinuric kidney disease has traditionally been approached exclusively from a glomerular perspective, but tubulointerstitial fibrosis is the hallmark histologic predictor of CKD progression. [12]. In this setting, kidney biopsy remains the gold standard for their diagnosis and evaluation [13]. However, it is an invasive procedure and tubulo-interstitial changes are detected when there is established damage, whereas tubular damage is an early feature of kidney insult. Consequently, numerous biomarkers of tubular health and disease are currently being evaluated to provide a comprehensive assessment of kidney health and disease [14]. Tubular biomarkers can be categorized into markers of tubular dysfunction, which assess the reabsorption capacity of low molecular weight proteins or the maintenance of tubular homeostasis [beta 2 microglobulin (β2-mcg), alpha 1 microglobulin (α1-mcg) and uromodulin (UMOD)]; and markers of tubular damage and tubulointerstitial repair, which quantify the severity of tubular damage and the extent of proinflammatory and fibrotic activity [neutrophil gelatinase-associated lipocalin (NGAL), kidney injury molecule-1 (KIM-1), monocyte chemoattractant protein-1 (MCP-1) and urinary Dickkopf-3 (uDKK3)] [15]. Several biomarkers have been evaluated as predictors of CKD progression and cardiovascular events in different population cohorts [16].

However, most tubular markers have not been specifically evaluated in patients with glomerular diseases and often lack histologic correlation. Proteinuria remains the only validated prognostic marker in most glomerular diseases but the prognostic value of proteinuria varies depending on the underlying disease [17] and there are patients who progress to ESKD despite the absence of high-grade proteinuria [18]. Therefore, there is a great need for biomarkers that predict eGFR loss in the high-risk group of proteinuric patients.

The aim of this study was to assess the levels of various tubular biomarkers in both serum and urine in patients with biopsied glomerular and proteinuric kidney disease, and to correlate them with kidney histology and kidney outcomes.

## MATERIALS AND METHODS

### Study design and population

A single-center retrospective observational study was conducted on a group of patients with proteinuric kidney diseases, who underwent kidney biopsy at the Gregorio Marañón University General Hospital between January 2016 and December 2021. This study was approved by the Medical Ethics Committee of HGUGM on 21 March 2022 and was conducted according to the Declaration of Helsinki.

Patients were included in the study if they were clinically stable and had persistent proteinuria (>150 mg in a 24-h urine collection) on at least two measurements separated by more than 1 month. Kidney biopsy was scheduled based on clinical indication. Exclusion criteria were acute kidney injury (AKI), estimated glomerular filtration rate (eGFR) by Chronic Kidney Disease Epidemiology Collaboration (CKD-EPI) <15 mL/min/1.73 m^2^, age <18 or >90 years, and loss or damage of serum or urine samples. AKI was defined as an increase in serum creatinine ≥0.3 mg/dL in the 48 h before kidney biopsy or an increase in serum creatinine ≥50% in the 7 days before the biopsy. AKI was excluded to avoid potential bias related to the transient increase in tubular marker levels during episodes of AKI.

Venous blood and urine samples obtained at the time of kidney biopsy were used to determine serum and urinary tubular markers.

### Clinical and laboratory evaluation

A systematic review of the electronic health record system, Health Care Information System (HCIS), was performed to collect demographic and laboratory data. All laboratory data were clinically obtained, not collected for the purpose of the study. GFR was estimated using the CKD-EPI 2021 equation.

### Tubular biomarkers

After obtaining informed consent, serum and urine samples were collected within a window of 1–12 h prior to kidney biopsy. These samples were aliquoted and stored at –80°C in the biobank, where they were labeled and recorded in the cohort database. These samples were sent to the Renal Pathophysiology Laboratory of the Gregorio Marañón Health Research Institute (IiSGM) for processing and determination of tubular markers. Biomarker analysis is summarized in the Supplementary data.

The following urinary and serum biomarkers were analyzed on the day of kidney biopsy: β2-mcg, α1-mcg, NGAL, urinary KIM-1 (uKIM-1), MCP-1, uDKK3, urinary UMOD (uUMOD), serum KIM-1 (sKIM-1) and serum UMOD (sUMOD).

Biomarker analysis and urine creatinine adjustment were blinded: laboratory personnel were unaware of clinical and histologic features. All urinary markers were standardized to urinary creatinine measured in the same urine sample to account for differences in urine concentration.

### Kidney histopathology

Kidney biopsies were evaluated by light microscopy and immunofluorescence by standard protocol (Supplementary data).

The following histologic data were collected, after clinical reading of pathology, for correlation with tubular biomarkers: date of kidney biopsy, glomerulosclerosis, percentage of glomeruli with glomerulosclerosis, tubular atrophy, percentage of tubular atrophy, interstitial fibrosis, percentage of interstitial fibrosis, degree of interstitial fibrosis [none (<10%), mild (10%–24%), moderate (25%–50%) and severe (>25%)], significant cortical interstitial inflammation (>10% of cortical parenchyma), arteriolar hyperinflation and degree of subintimal fibrosis. Interstitial fibrosis was analyzed as a continuous and categorical variable.

### Kidney and mortality outcomes

Annual decline in eGFR and changes in proteinuria/albuminuria were assessed by systematic review of the patients’ electronic medical records during the follow-up.

Kidney function was evaluated using the CKD-EPI eGFR equation for creatinine (mL/min/1.73 m^2^). Proteinuria was determined in 24-h urine samples using the hospital laboratory's reference nephelometric methods. Progression of CKD or kidney event were defined as a decrease in eGFR ≥40% from baseline or the development of ESKD (eGFR ≤10 mL/min or initiation of kidney replacement therapy). Mortality events were recorded, including the cause of death (cardiovascular, neoplastic, infectious or other cause).

The primary outcome was a composite of kidney event or death. Participants were censored at death or at the date of the last available follow-up information at the time of data collection for the study.

### Statistical analysis

Statistical analysis was performed using the SPSS Statistics program, version 25 (SPSS, Inc., Chicago, IL, USA). Quantitative variables were expressed as median and interquartile range. The Kolmogorov–Smirnov test was used to determine whether quantitative variables followed a normal distribution. Variables with non-normality distribution were log transformed. Percentages and absolute values were used for qualitative variables. Categorical variables were compared using the χ² (chi-squared) test and Fisher's exact test. Medians were compared using nonparametric tests (Mann–Whitney U test for independent samples and Kruskal–Wallis test).

The Spearman correlation coefficient was used to assess the association between quantitative variables. Multivariate linear regression analysis (enter model) was used to independently evaluate predictive factors for specific histologic parameters. In order to assess the discriminative ability of the markers, a receiver operating characteristic (ROC) curve analysis was performed to estimate the area under the curve (AUC). Logistic regression analysis was used to estimate the odds ratio (OR) for the presence of specific histologic lesions based on tubular markers.

Linear mixed-effect model was used to evaluate the association of each biomarker with eGFR decline. Cox regression analysis was used to estimate the relative risk of kidney events or mortality based on the biomarkers, adjusted for other clinical or histologic variables. For kidney outcomes, we performed a sensitivity analysis for the competing risk of death. Kaplan–Meier curves were also used to compare event-free kidney survival among different biomarker tertiles. Multivariate models were adjusted for sociodemographic characteristics (age, sex, body mass index), hypertension, diabetes, smoking, eGFR, proteinuria and interstitial fibrosis. We performed a change-in-estimate method to identify the variable whose exclusion resulted in the smallest change in the outcome association. By removing these variables, we narrowed the adjustment to age, eGFR, proteinuria and interstitial fibrosis. Statistical significance was set at *P* < .05.

## RESULTS

### Baseline data

**Figure 1:**
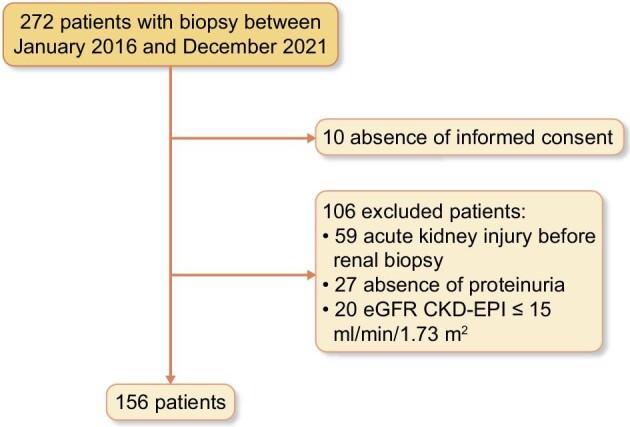
Flow chart of the study.

During the study period, 272 kidney biopsies were performed, of which 156 patients met the inclusion criteria and were enrolled in the study. The study flow chart is shown in Fig. 1.

Regarding the etiology of proteinuric kidney disease, glomerular disease accounted for the majority (68.6%), followed by diabetic kidney disease (12.8%). Clinical, analytical and histological characteristics are summarized in Table 1.

**Table 1:** Baseline characteristics.

Baseline characteristics	
Age (years)	54.1 ± 15.9
Male, *n* (%)	89 (57.1)
Ethnicity, *n* (%)	
African	6 (3.8)
Asiatic	5 (3.2)
Caucasian	114 (73.1)
Latin American	31 (19.9)
Hypertension, *n* (%)	114 (73.1)
Dyslipidemia, *n* (%)	96 (61.5)
Diabetes mellitus, *n* (%)	38 (24.4)
Obesity, *n* (%)	26 (16.7)
Smoking, *n* (%)	28 (17.9)
Cardiovascular disease, *n* (%)	48 (30.8)
CKD etiology, *n* (%)	
Glomerular	107 (68.6)
Membranous nephropathy	16 (10.3)
IgA nephropathy	16 (10.3)
Lupus nephritis	15 (9.6)
FSGS	12 (7.7)
Minimal change disease	12 (7.7)
MPGN	8 (5.1)
Amyloidosis	8 (5.1)
Others	7 (4.5)
ANCA vasculitis	7 (4.5)
TMA/aHUS	6 (3.8)
Diabetic kidney disease, *n* (%)	20 (12.8)
Mixed, *n* (%)	6 (3.8)
Interstitial, *n* (%)	4 (2.6)
Vascular, *n* (%)	3 (1.9)
Obstructive, *n* (%)	1 (0.6)
Others, *n* (%)	15 (9.6)
Serum creatinine (mg/dL)	1.6 (1.0, 2.1)
eGFR (mL/min/1.73 m^2^)	42.5 (29.3, 77.3)
Proteinuria (mg/24 h)	2387 (972, 5562)
Nephrotic syndrome, *n* (%)	47 (30.1)
Microhematuria, *n* (%)	112 (71.8)
RAAS blockers	124 (79.5)
Diuretics	78 (50)
iSGLT2	12 (7.7)

Data are presented as mean standard deviation, median (interquartile range) or *n* (%).

### Associations between tubular biomarkers and clinical data

Median levels of tubular biomarkers were determined at different stages of CKD and at different levels of proteinuria. Higher levels of proteinuria were associated with increased levels of β2-mcg and α1-mcg levels, and low levels of uUMOD and sUMOD (Supplementary data, Tables S1 and S2).

### Associations between tubular biomarkers and histology

**Figure 2:**
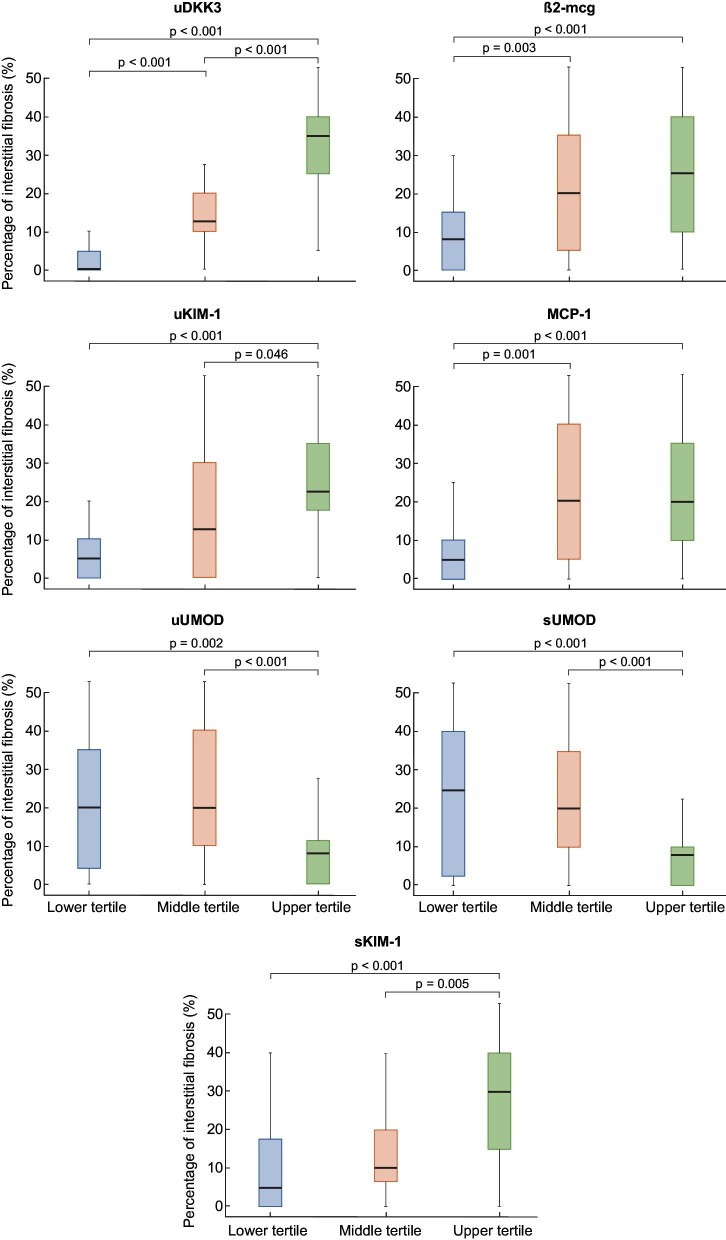
Box-plot distribution of median interstitial fibrosis based on tertiles of urinary biomarkers. **Kruskal–Wallis *P*-value, significant for differences among at least one group if *P* <.05.

A correlation was found between the extent of interstitial fibrosis in the biopsy and certain biomarkers (Supplementary data, Table S3). uDKK3 showed the strongest correlation with fibrosis, which persisted after adjustment for proteinuria and eGFR (Supplementary data, Table S4). Median interstitial fibrosis based on tertiles of urinary biomarkers is shown in Fig. 2.

Multivariate regression analysis identified uDKK3 as a strong and independent predictor of interstitial fibrosis (β 0.483, *P* < .001) adjusted for age, eGFR, log proteinuria and other tubular markers, with a corrected R^2^ of 0.687 (Supplementary data, Table S5). In the same model, sUMOD (β –0.357, *P* < .001) and sKIM-1 (β 0.183, *P* = .036) also showed a significant, but weaker, correlation with interstitial fibrosis.

The discriminative ability of different tubular markers to predict interstitial fibrosis >25% in the biopsy was examined using ROC curve analysis (Supplementary data, Fig. S1 and Table S6). uDKK3 showed the highest AUC [0.918, 95% confidence interval (CI) 0.866–0.971, *P* < .001], although β2-mcg, uKIM-1, MCP-1, uUMOD, sKIM-1 and sUMOD also had statistically significant AUCs.

Patients with cortical interstitial inflammation >10% presented higher levels of β2-mcg, uKIM-1, MCP-1, uDKK3 and sKIM-1, and lower levels of uUMOD (Supplementary data, Fig. S2).

Elevated MCP-1 levels were associated with higher risk of cortical interstitial inflammation >10% in the logistic regression analysis adjusted for age, eGFR, log proteinuria and microhematuria: MCP-1 OR 15.61, 95% CI 3.52–69.20, *P* < .001 (Table 2).

**Table 2:** Logistic regression analysis for cortical interstitial inflammation >10% adjusted for log uKIM-1 and log MCP-1, age, microhematuria, log proteinuria and eGFR.

	OR	95% CI
Age	0.975	0.937–1.014
Microhematuria	0.242	0.056–1.044
Log proteinuria (mg)	0.804	0.448–1.440
eGFR (mL/min/1.73 m^2^)	0.970	0.943–0.998
Log uKIM-1 (µg/mg)	3.150	0.948–10.464
Log MCP-1 (µg/mg)	15.605	3.519–69.198

Tubular biomarkers were adjusted for age, microhematuria, log proteinuria and eGFR.

There were no other significant correlation between tubular markers and hyalinosis or myointimal fibrosis.

### Associations of tubular biomarkers with kidney and mortality outcomes

Patients were followed for a median time of 26 (12.5, 41.4) months after kidney biopsy.

**Figure 3:**
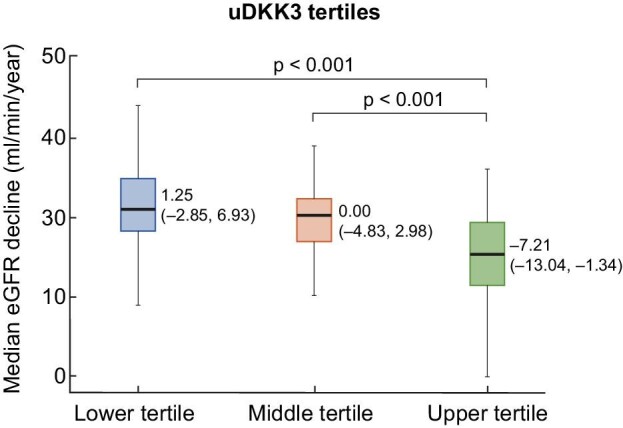
Box-plot distribution of eGFR decline by uDKK3 tertiles. **Kruskal–Wallis *P*-value, significant for differences among at least one group if *P* < .05 (B).

A linear mixed-effect model was performed to evaluate the association of each biomarker with eGFR decline and found a negative and significant association only for uDKK3. Each one unit increase in uDKK3 was associated with a –0.37 (95% CI –0.86 to 0.12) mL/min/1.73 m^2^/year decline in eGFR (*P* < .001) (Supplementary data, Table S7). Moreover, the upper tertile of uDKK3 was associated with a greater decline in eGFR [–7.21 (–13.04, –1.34) mL/min/1.73 m^2^/year] compared with the middle and lower tertiles (Fig. 3).

Cox regression analysis was used to assess the risk of the composite outcome based on tubular biomarkers. Per doubling uDKK3 showed a hazard ratio (HR) of 5.50 (95% CI 3.01–10.10) for the composite event, but was not statistically significant after adjustment for age, eGFR, log proteinuria and interstitial fibrosis (HR 1.72, 95% CI 0.85–3.48) (Table 3).

**Table 3:** Univariate and multivariate Cox regression analysis for composite outcome per doubling of uDKK3 adjusted for age, log proteinuria, eGFR and interstitial fibrosis.

	HR	95% CI
Univariate analysis		
uDKK3 (per doubling)	5.502	3.001–10.087
Multivariate analysis		
Age (years)	0.996	0.976–1.017
Log proteinuria (mg)	1.650	1.166–2.334
eGFR^a^ (mL/min/1.73 m^2^)	0.962	0.942–0.983
Interstitial fibrosis	1.032	1 009–1.056
uDKK3 (per doubling)	1.724	0.853–3.483

a eGFR by CKD-EPI equation for creatinine, mL/min/1.73 m^2^.

b HR per doubling increase in uDKK3.

**Figure 4:**
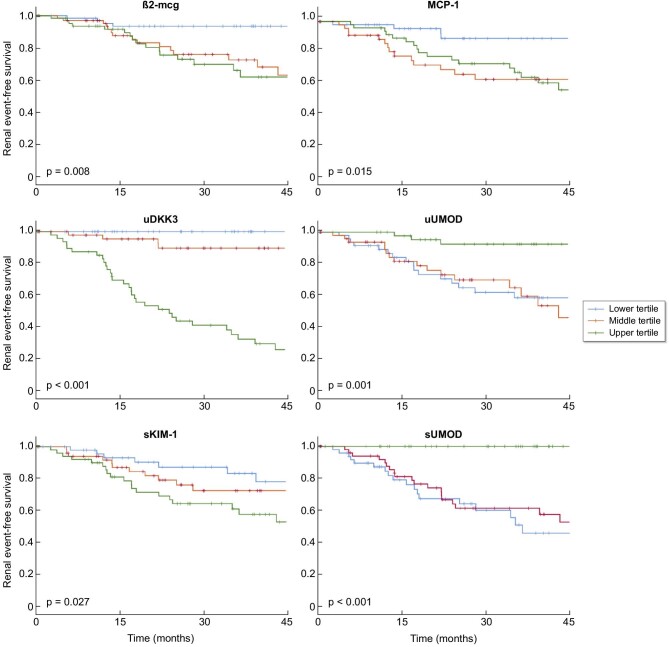
Kidney event-free survival based on tertiles of tubular markers **P, Log Rank (Mantel–Cox).

During the observation period, there were a total of 41 kidney events (26.3%) and 25 patients (16%) required initiation of kidney replacement therapy. It was observed that patients with higher tertiles of β2-mcg, MCP-1, uDKK3 and KIM-1s, and lower tertiles of uUMOD and sUMOD had a significantly reduced kidney event-free survival in the Kaplan–Meier model analysis (Fig. 4). Each doubling of uDKK3 was associated with a 2.2-fold increased risk of a kidney event (HR 2.26, 95% CI 1.04–4.94) after adjustment for age, eGFR, log proteinuria and interstitial fibrosis (Supplementary data, Table S8).

The mortality rate was 12.8% (20 deaths), with neoplastic disease being the primary cause (50%). Per doubling uDKK3 was associated with a higher risk of mortality (HR 3.65, 95% CI 1.26–10.55), but was not confirmed in the multivariate model adjusted for age, log proteinuria, eGFR and interstitial fibrosis (Supplementary data, Table S7).

No other tubular marker was associated with kidney event or death.

## DISCUSSION

This study found that certain tubular biomarkers provide additional information in the assessment of proteinuric kidney disease, correlating with specific histologic findings.

Distribution of tubular markers was consistent with published literature [19, 20]. Higher levels of proteinuria were significantly correlated with an increase in urinary proteins typically reabsorbed by the tubule (β2-mcg and α1-mcg), while others secreted under physiological conditions decreased (UMOD). These association trends have been previously reported for β2-mcg and α1-mcg [21], but not for uUMOD, supporting the role of proteinuria as a direct contributor to tubular dysfunction.

Tubular atrophy and interstitial fibrosis are recognized as key factors in kidney prognosis [12], although several studies have demonstrated their presence in a significant number of patients without a concomitant decrease in eGFR [22]. Our study demonstrated a correlation between the extent of interstitial fibrosis in the biopsy and the tubular markers uDKK3, uKIM-1, sKIM-1, MCP-1, β2-mcg, uUMOD and sUMOD, as previously demonstrated for some of these markers in other studies, but not specifically in proteinuric diseases [23–25].

Among these, uDKK3 showed the strongest correlation and emerged as a predictor of interstitial fibrosis independent of eGFR and proteinuria. uDKK3 is a Dickkopf family glycoprotein secreted by tubular cells in response to stress conditions and functions by activating the Wnt/β-catenin signaling pathway and ultimately promoting interstitial fibrosis [26, 27]. Another study evaluating this marker in patients with glomerular or interstitial diseases reported that elevated levels of uDKK3 were associated with a greater extent of interstitial fibrosis [25]. sUMOD also proved to be an inverse predictor of interstitial fibrosis in the model, although weaker than uDKK3. This finding has been observed in other studies where elevated levels of UMOD were associated with a lower degree of tubulointerstitial fibrosis in both human kidney biopsies and mouse models [24].

Another histologic parameter examined was cortical interstitial inflammation, a process that, if sustained, ultimately predisposes to the development of irreversible interstitial fibrosis and is a widely recognized risk factor for kidney progression [3]. In our study, elevated levels of MCP-1 were associated with a higher risk of significant cortical interstitial inflammation, independent of eGFR, proteinuria and microhematuria. MCP-1 is secreted by proximal tubular cells in response to proinflammatory situations and are involved in processes of regeneration and repair of the tubular epithelium [14]. Their association with cortical interstitial inflammation has been demonstrated in biopsy specimens from patients with lupus nephritis, kidney transplantation and even diabetic nephropathy [28–31], but not specifically assessed in patients with proteinuric disease. Thus, the use of these tubular markers could help us predict the results of kidney biopsy.

The prediction of eGFR loss during long-term follow-up is a critical aspect in the management of CKD patients, and is currently attempted by the use of kidney failure risk equations that include baseline eGFR, proteinuria and laboratory and clinical factors such as hypertension or diabetes [32]. However, given the considerable variability in the trajectory of individual eGFR [33], there is a need for alternative prognostic tubular markers. Upper tertiles of β2-mcg/creatinine, MCP-1, uDKK3 and sKIM-1, and lower tertiles of uUMOD and sUMOD were associated with an increased risk of kidney events. The prognostic utility of these markers has been previously demonstrated in studies conducted in different CKD population cohorts [14, 23, 34–38]. However, our analysis was specifically performed on high-grade proteinuric patients (up to 37% in the nephrotic range) and included the assessment of multiple tubular markers.

Notably, each increase and higher tertile of uDKK3 was associated with greater eGFR decline. Although it was not a predictor of the composite outcome, it was associated with kidney events after adjustment for age, eGFR, proteinuria and interstitial fibrosis. In previous studies in patients with CKD of various etiologies, elevated baseline uDKK3 levels were associated with an increased risk of CKD progression, independent of the underlying etiology and the levels of creatinine and albuminuria [25, 39]. In addition, in patients with immunoglobulin A nephropathy from the STOP-IgA cohort, an increase in uDKK3 levels at 6 months was associated with a greater decline in eGFR, whereas stabilization or reduction of these levels indicated a more favorable prognosis [40]. In our study, uDKK3 could help identify patients at higher risk in a highly proteinuric population prone to kidney disease progression.

uDKK3 was associated with increased mortality in univariate analysis but not in the multivariate model, probably due to the influence of covariates and the limited sample size. There are published data on its prognostic role in various types of cancer: gastric, gynecologic, colorectal, hematologic and renal [41–43]. It has been observed that DKK3 may play a cardioprotective role by preventing ventricular dysfunction and cardiac remodeling after myocardial infarction [44].

This study is not without limitations: it is retrospective and single-center, and the sample size is limited with different etiologies included, which may lead to a bias in the overall analysis. There is a selection bias in the study sample based on indications for biopsy. In addition, changes in these markers over time were not measured and it is a cross-sectional study at the time of the biopsy. On the other hand, it also has strengths: for the first time, seven tubular markers were analyzed together and correlated with histological changes and kidney events specifically in proteinuric diseases. It will be of great interest to assess whether there are changes in these tubular markers in response to specific treatments that modify proteinuria and/or the rate of CKD progression.

In summary, uDKK3 and sUMOD were predictors of interstitial fibrosis and MCP-1 was a predictor of interstitial inflammation. uDKK3 was not a predictor of the composite outcome, but it was associated with kidney events, even after adjustment for interstitial fibrosis, eGFR and proteinuria. Thus, specific tubular markers and their combination have the potential to predict renal histology and provide additional insight into kidney prognosis.

## Supplementary Material

sfae146_Supplemental_File

## Data Availability

The data sets analyzed in the current study are available upon request from the corresponding author.
