# Supplementary material for: Tubular biomarkers in proteinuric kidney disease: histology correlation and kidney prognosis of tubular biomarkers
Source: Clin Kidney J. 2024 May 9;17(5):sfae146. doi: 10.1093/ckj/sfae146 (PMC11129590; doi:10.1093/ckj/sfae146)
Supplement: sfae146_Supplemental_File [file sfae146_supplemental_file.docx]

**SUPPLEMENTARY MATERIAL.**

| **Supplementary Data** | Tubular biomarker analysis and kidney histopathology assesment |
| --- | --- |
| **Table S1** | Median and comparison of medians among tubular markers based on the stage of chronic kidney disease (CKD). |
| **Table S2** | Median and comparison of medians among tubular markers based on the degree of proteinuria. |
| **Table S3** | Correlation of tubular biomarker with interstitial fibrosis. |
| **Table S4** | Correlation between tubular markers and interstitial fibrosis after adjustment for eGFR and proteinuria. |
| **Table S5** | Multivariate regression analysis for the percentage of interstitial fibrosis. |
| **Table S6** | Area under the curve (AUC) for ROC curves to predict interstitial fibrosis >25% based on tubular markers |
| **Table S7** | Linear mixed-effect model of estimated eGFR decline for each tubular biomarker. |
| **Table S8** | Univariate and multivariate Cox regression analysis for kidney event. |
| **Table S9** | Univariate and multivariate Cox regression analysis for mortality event |
| **Figure S1** | ROC curves for predicting interstitial fibrosis >25% in the biopsy based on levels of tubular biomarkers. |
| **Figure S2** | Values of tubular markers according to the degree of cortical interstitial inflammation in the biopsy |

**Supplementary Data.**

**Tubular biomarker.**

The concentration of the markers in urine and serum was determined using specific colorimetric enzyme-linked immunosorbent assay (ELISA) kits for each of them.

The following urinary markers, adjusted to urine creatinine were analyzed:

- Beta 2 microglobulin (β2-mcg). Human β-2 Microglobulin ELISA Kit.
- Alpha 1 microglobulin (α1-mcg). Alpha-1-Microglobulin/Bikunin Precursor (a1M) (Cloud-Clone Corp., TX, USA).
- NGAL (Neutrophil Gelatinase-Associated Lipocalin). Human NGAL Sandwich ELISA Kit (Proteintech, IL, USA).
- uKIM-1 (urine Kidney Injury Molecule-1). Human KIM-1/HAVCR1 Sandwich ELISA Kit (Proteintech, IL, USA).
- MCP-1 (Monocyte Chemoattractant Protein-1). Human MCP-1/CCL2 ELISA Kit (Alpha Diagnostic Intl Inc., TX, USA).
- uDKK-3 (urinary Dickkopf-3). Human Dkk-3 ELISA Kit (Sigma-Aldrich, MO, USA).
- uUMOD (urine uromodulin). Human UMOD(Uromodulin) ELISA Kit (MyBiosource, CA, USA).

Serum biomarkers:

- sKIM-1 (serum Kidney Injury Molecule-1). Human KIM-1(Kidney Injury Molecule 1) ELISA Kit (Elabscience, TX, USA).
- sUMOD (serum uromodulin). Human UMOD(Uromodulin) ELISA Kit (MyBiosource, CA, USA).

The concentration of each sample was quantified by interpolating the absorbance reading on a standard curve generated with the standards provided with the specific kit and expressed in the appropriate units. The Synergy™ HT microplate reader or spectrophotometer (BioTek® Instruments, Inc., VE, USA) with the Gen5™ program was used for absorbance readings. The MyAssays online platform was used to analyze the results.

**Kidney Histopathology.**

Standard processing protocols for kidney samples were performed by the Department of Pathology. For light microscopy, the included sections were routinely stained with hematoxylin and eosin (H&E), periodic acid-Schiff (PAS), Masson's trichrome, and silver methenamine. Immunofluorescence staining for IgG, IgA, IgM, C3, and C1q antisera was performed on frozen kidney specimens.

| **Kidney histology** | |
| --- | --- |
| Glomerular sclerosis, n (%)  Percentage of glomerular sclerosis (%)  Degree of glomeruloesclerosis  < 25%  25-50%  > 50%  Tubular atrophy, n (%)  Percentage of tubular atrophy (%)  Interstitial fibrosis, n (%)  Percentage of interstitial fibrosis (%)  Degree of interstitial fibrosis  < 25%, n (%)  25-50%, n (%)  > 50%, n (%)  Cortical interstitial inflammation > 10%, n (%)  Arteriolar hyalinosis, n (%)  Degree of myointimal fibrosis, n (%)  < 25%  25-50%  >50% | 116 (74.4%)  15.1 (0, 33)  99 (63.5%)  42 (26.9%)  15 (9.6%)  90 (57.7%)  10 (0, 30)  113 (72.4%)  15 (5, 30)  107 (68.6%)  41 (26.3%)  8 (5.1%)  54 (34.6%)  58 (37.2%)  127 (81.4%)  23 (14.7%)  5 (3.2%) |

**Table S1.** Median and comparison of medians among tubular markers based on the stage of chronic kidney disease (CKD).

| CKD stage (eGFR)  ml/min/1.73 m2 | **β2-mcg**  **mcg/mg** | **α1-mcg**  **mcg/mg** | **NGAL**  **mcg/mg** | **uKIM-1**  **mcg/mg** | **MCP-1**  **mcg/mg** | **uDKK3**  **mcg/mg** | **uUMOD**  **ng/mg** | **sKIM-1**  **pg/ml** | **sUMOD**  **ng/ml** |
| --- | --- | --- | --- | --- | --- | --- | --- | --- | --- |
| > 90 (n=29) | 0,2 (0,1;1,2) | 8 (2,7; 15,8) | 24,3 (14,8; 15,6) | 1,15 (0,7; 1,8) | 0,6 (0,3; 1,1) | 1,2 (0,6; 2,3) | 4,4 (1; 5,3) | 0 (0; 1,6) | 43,3 (13,5; 54,2) |
| 60-90 (n=24) | 0,2 (0; 2,4) | 4,8 (1,6; 11,4) | 17,8 (1,5; 49) | 1,3 (0,7; 3) | 0,8 (0,2; 1,3) | 1,1 (0,5; 1,7) | 3,8 (1; 5,2) | 0 (0; 1,8) | 32,6 (7,7; 66,2) |
| 45-60 (n=22) | 2,7 (0,3; 9,9) | 6,1 (4,5; 14) | 35,9 (10; 67) | 1,9 (1,1; 3,8) | 1,9 (0,9; 2,5) | 4,4 (0,9; 15) | 2,4 (0,6; 4,5) | 3,8 (0; 6,5) | 10,6 (2; 38,2) |
| 30-45 (n=41) | 1,9 (0,1; 10,6) | 4,5 (1,4; 11) | 45,7 (15,6; 69,8) | 2,2 (1,2; 4,2) | 1,5 (0,9; 2,5) | 6,2 (2,3; 19,6) | 2,3 (1,1; 3,6) | 7,1 (1,9; 10,1) | 15 (5,1; 42,2) |
| < 30 (n=40) | 4,7 (1,2; 11,2) | 4 (2,6; 9) | 0 (0; 40,7) | 3,3 (1,9; 4,6) | 2,2 (1,4; 3,2) | 19,7 (11,2; 37,1) | 1,1 (0,2; 2,3) | 8,8 (3,9; 17,3) | 13 (2,2; 22,5) |
| p | **< 0,001** | 0,45 | 0,25 | **< 0,001** | **< 0,001** | **< 0,001** | **0,001** | **< 0,001** | **0,015** |

*β2-mcg, beta 2 microglobulin; α1-mcg, alpha 1 microglobulin; NGAL, Neutrophil Gelatinase-Associated Lipocalin; uKIM-1, urine Kidney Injury Molecule-1 urine; MCP-1, Monocyte Chemoatractant Protein 1; uDKK3, urinary Dickkopf-3; uUMOD, urinary uromodulin; sKIM-1, serum Kidney Injury Molecule-1; sUMOD, serum uromodulin.

** Kruskal-Wallis p-value, significant for differences among at least one group if p <0.05.

**Table S2.** Median and comparison of medians among tubular markers based on the degree of proteinuria.

| Proteinuria  (mg/24h) | **β2-mcg**  **mcg/mg** | **α1-mcg**  **mcg/mg** | **NGAL**  **mcg/mg** | **uKIM-1**  **mcg/mg** | **MCP-1**  **mcg/mg** | **uDKK3**  **mcg/mg** | **uUMOD**  **ng/mg** | **sKIM-1**  **pg/ml** | **sUMOD**  **ng/ml** |
| --- | --- | --- | --- | --- | --- | --- | --- | --- | --- |
| < 300 (n=6) | 0,2 (0,1; 2) | 1,8 (0,2; 3,9) | 36,6 (28,7; 61,2) | 2,8 (1,1; 4,3) | 2,3 (0,6; 3,8) | 0,5 (0,2; 3,9) | 6,3 (5; 7,7) | 1,5 (0; 8,2) | 49,6 (46,2; 62,2) |
| 300-1000 (n=36) | 0,3 (0,1; 2,1) | 1,5 (0,5; 2,6) | 29,3 (6,6; 60,8) | 1,7 (0,9; 3,3) | 0,9 (0,4; 2,2) | 2,3 (0,6; 5,6) | 4 (2,7; 5) | 3,6 (0,1; 8,4) | 41,6 (29,1; 62,5) |
| 1000-3500 (n=56) | 0,6 (0,1; 5) | 4,8 (2,4; 9,1) | 18,1 (0; 47,6) | 1,8 (1; 3,4) | 1,1 (0,6; 2,4) | 7,4 (1,2; 24,3) | 2,7 (1,2; 4,5) | 3 (0; 10) | 20,8 (9; 45,5) |
| > 3500 (n=58) | 4,8 (1,6; 17,7) | 11,5 (6,1; 18,7) | 31 (9; 65) | 2,5 (1,2; 4,1) | 1,6 (1; 2,5) | 5,8 (1,5; 18,2) | 0,8 (0,2; 1,4) | 5,2 (0; 8,9) | 7,2 (1,4; 11,5) |
| P | **< 0,001** | **< 0,001** | 0,173 | 0,278 | 0,118 | 0,010 | **< 0,001** | 0,747 | **< 0,001** |

*β2-mcg, beta 2 microglobulin; α1-mcg, alpha 1 microglobulin; NGAL, Neutrophil Gelatinase-Associated Lipocalin; uKIM-1, urine Kidney Injury Molecule-1 urine; MCP-1, Monocyte Chemoatractant Protein 1; uDKK3, urinary Dickkopf-3; uUMOD, urinary uromodulin; sKIM-1, serum Kidney Injury Molecule-1; sUMOD, serum uromodulin.

** Kruskal-Wallis p-value, significant for differences among at least one group if p <0.05.

**Table S3. Correlation of tubular biomarker with interstitial fibrosis.**

| Tubular biomarkers | Rho | p |
| --- | --- | --- |
| β2-mcg (mcg/mg) | 0.392 | **< 0.001** |
| α1-mcg (mcg/mg) | 0.102 | 0.452 |
| NGAL (mcg/mg) | 0,058 | 0.891 |
| uKIM-1 (mcg/mg) | 0.368 | **<0.002** |
| MCP-1 (mcg/mg) | 0.374 | **<0,001** |
| uDKK3 (mcg/mg) | 0.804 | **< 0.001** |
| uUMOD (ng/mg) | 0.317 | **< 0.001** |
| sKIM-1 (pg/ml) | 0.472 | **< 0.001** |
| sUMOD (ng/ml) | 0.370 | **< 0.001** |

*β2-mcg, beta 2 microglobulin; α1-mcg, alpha 1 microglobulin; NGAL, Neutrophil Gelatinase-Associated Lipocalin; uKIM-1, urine Kidney Injury Molecule-1 urine; MCP-1, Monocyte Chemoatractant Protein 1; uDKK3, urinary Dickkopf-3; uUMOD, urinary uromodulin; sKIM-1, serum Kidney Injury Molecule-1; sUMOD, serum uromodulin.

**Table S4.** Correlation between tubular markers and interstitial fibrosis after adjustment for eGFR and proteinuria.

|  | **β2mcg/Cro** | **KIM1o/Cro** | **MCP1/Cro** | **uDKK3/Cro** | **UMODo/Cro** | **KIM1s** | **UMODs** |
| --- | --- | --- | --- | --- | --- | --- | --- |
| **Adjusted for proteinuria levels** | | | | | | | |
| Proteinuria < 1  (n=42) | 0,295  p=0,065 | 0,339  p=0,032 | 0,437  **p=0,005** | 0,707  **p<0,001** | -0,416  **p=0,008** | 0,465  **p<0,001** | -0,391  **p=0,010** |
| Proteinuria 1 - 3,5  (n=56) | 0,552  **p<0,001** | 0,471  **p=0,012** | 0,492  **p<0,001** | 0,804  **p<0,001** | -0,614  **p<0,001** | 0,541  **p<0,001** | -0,545  **p<0,001** |
| Proteinuria > 3,5  (n=58) | 0,343  **p<0,010** | 0,350  **p<0,001** | 0,315  **p=0,018** | 0,818  **p<0,001** | -0,176  p=0,194 | 0,438  **p=0,001** | -0,306  **p=0,019** |
| **Adjusted for eGFR** | | | | | | | |
| eGFR > 60  (n=53) | -0,033  p=0,822 | -0,003  p=0,982 | 0,079  p=0,584 | 0,432  **p=0,002** | -0,044  p=0,761 | 0,051  p=0,716 | 0,003  p=0,982 |
| eGFR < 60  (n=103) | 0,319  **p=0,001** | 0,119  p=0,242 | 0,066  p=0,532 | 0,704  **p<0,001** | -0,355  **p<0,001** | 0,346  **p<0,001** | -0,426  **p<0,001** |

* β2-mcg, beta 2 microglobulin; uKIM-1, urine Kidney Injury Molecule-1 urine; MCP-1, Monocyte Chemoatractant Protein 1; uDKK3, urinary Dickkopf-3; uUMOD, urinary uromodulin; sKIM-1, serum Kidney Injury Molecule-1; sUMOD, serum uromodulin; eGFR, estimated glomerular filtration rate.

**p, non-parametric correlation coefficient, Spearman's rho

**Table S5.** Multivariate regression analysis for the percentage of interstitial fibrosis. Independent variables in model 1: log β2-mcg, log uKIM-1, log MCP-1, log uDKK3, log uUMOD, log sKIM-1, and log sUMOD. Independent variables in model 2 adjusted for age, log proteinuria and serum creatinine: log β2-mcg, log uKIM-1, log MCP-1, log uDKK3, log uUMOD, log sKIM-1, and log sUMOD.

| **Model 1** | **Standardized coefficients**  **Beta** | **p** |
| --- | --- | --- |
| (Constant) |  | **<0,001** |
| Log β2-mcg (mcg/mg) | 0,045 | 0,663 |
| Log uKIM-1 (mcg/mg) | -0,138 | 0,227 |
| Log MCP-1 (mcg/mg) | 0,087 | 0,443 |
| Log uDKK3 (mcg/mg) | 0,522 | **<0,001** |
| Log uUMOD (ng/mg) | 0,168 | 0,099 |
| Log sKIM-1 (pg/ml) | 0,234 | **0,008** |
| Log sUMOD (ng/ml) | -0,340 | **0,001** |
| *Corrected R^2^* | *0,626* | |
| **Model 2** | **Standardized coefficients**  **Beta** | **p** |
| (Constant) |  | <0,001 |
| Age (years) | -0,099 | 0,205 |
| eGFR (ml/min/1.73m2) | -0,199 | 0,040 |
| Log proteinuria (mg/24h) | -0,200 | 0,041 |
| Log β2-mcg (mcg/mg) | 0,054 | 0,595 |
| Log uKIM-1 (mcg/mg) | -0,155 | 0,151 |
| Log MCP-1 (mcg/mg) | 0,060 | 0,583 |
| **Log uDKK3 (mcg/mg)** | **0,483** | **<0,001** |
| Log uUMOD (ng/mg) | 0,069 | 0,507 |
| Log sKIM-1 (pg/ml) | **0,183** | **0,036** |
| **Log sUMOD (ng/ml)** | **-0,357** | **<0,001** |
| *Corrected R^2^* | *0,687* | |

*β2-mcg, beta 2 microglobulin; uKIM-1, urine Kidney Injury Molecule-1 urine; MCP-1, Monocyte Chemoatractant Protein 1; uDKK3, urinary Dickkopf-3; uUMOD, urinary uromodulin; sKIM-1, serum Kidney Injury Molecule-1; sUMOD, serum uromodulin; GMR; geometric mean ratio

**Table S6.** Area under the curve (AUC) for ROC curves to predict interstitial fibrosis >25% based on tubular markers

| Tubular biomarkers | AUC | IC 95% | p |
| --- | --- | --- | --- |
| β2-mcg (mcg/mg) | 0,733 | 0,652-0,813 | **< 0,001** |
| α1-mcg (mcg/mg) | 0,489 | 0,388-0,859 | 0,826 |
| NGAL (mcg/mg) | 0,439 | 0,335-0,543 | 0,243 |
| uKIM-1 (mcg/mg) | 0,658 | 0,564-0,752 | **0,002** |
| MCP-1 (mcg/mg) | 0,669 | 0,582-0,757 | **0,001** |
| uDKK3 (mcg/mg) | 0,918 | 0,866-0,971 | **< 0,001** |
| uUMOD (ng/mg) | 0,260 | 0,178-0,342 | **< 0,001** |
| sKIM-1 (pg/ml) | 0,787 | 0,701-0,873 | **< 0,001** |
| sUMOD (ng/ml) | 0,236 | 0,159-0,312 | **< 0,001** |

*β2-mcg, beta 2 microglobulin; α1-mcg, alpha 1 microglobulin; NGAL, Neutrophil Gelatinase-Associated Lipocalin; uKIM-1, urine Kidney Injury Molecule-1 urine; MCP-1, Monocyte Chemoatractant Protein 1; uDKK3, urinary Dickkopf-3; uUMOD, urinary uromodulin; sKIM-1, serum Kidney Injury Molecule-1; sUMOD, serum uromodulin.

**Table S7.** Linear mixed-effect model of estimated eGFR decline for each tubular biomarker.

| Biomarker | Estimated eGFR coefficient | SD | 95% CI | p |
| --- | --- | --- | --- | --- |
| β2-mcg (mcg/mg) | -0.03 | 0.06 | -0.16 – 0.08 | 0.53 |
| α1-mcg (mcg/mg) | 0.36 | 8.51 | -15.21 – 15.65 | 1.00 |
| NGAL (mcg/mg) | -0.02 | 0.01 | -0.03 – 0.03 | 0.90 |
| uKIM-1 (mcg/mg) | -0.82 | 10.8 | -43.3 – 40.62 | 1.00 |
| MCP-1 (mcg/mg) | 0.26 | 8.25 | - 15.91 – 16.44 | 0.97 |
| **uDKK3 (mcg/mg)** | **-0.37** | **0.17** | **-0.86 – 0.12** | **< 0.001** |
| uUMOD (ng/mg) | 1.41 | 0.80 | -0.16 – 2.99 | 0.08 |
| sKIM-1 (pg/ml) | -0.33 | 12.02 | -23.90 – 23.23 | 0.98 |
| sUMOD (ng/ml) | 0.18 | 11.7 | -22.86 – 23.22 | 0.99 |

*β2-mcg, beta 2 microglobulin; α1-mcg, alpha 1 microglobulin; NGAL, Neutrophil Gelatinase-Associated Lipocalin; uKIM-1, urine Kidney Injury Molecule-1 urine; MCP-1, Monocyte Chemoatractant Protein 1; uDKK3, urinary Dickkopf-3; uUMOD, urinary uromodulin; sKIM-1, serum Kidney Injury Molecule-1; sUMOD, serum uromodulin; eGFR, estimated glomerular filtration rate (ml/min/1.73m2).

** Estimated eGFR coefficient indicates the estimated change in eGFR associated with a one-unit increase for each biomarker.

**Table S8**. Univariate and multivariate Cox regression analysis for kidney event for per doubling of uDKK3 adjusted for age, log proteinuria, eGFR and interstitial fibrosis.

|  | HR | CI (95%) |
| --- | --- | --- |
| **Univariate analysis** |  |  |
| uDKK3 (per doubling) | 7.463 | 3.811-14.613 |
| **Multivariate analysis** |  |  |
| Age (years) | 0.987 | 0.963-1.011 |
| Log proteinuria (mg) | 1.972 | 1.283-3.032 |
| eGFR (ml/min/1.73m2) | 0.952 | 0.926-0.978 |
| Interstitial fibrosis | 1.035 | 1.008-1.062 |
| uDKK3 (per doubling) | 2.264 | 1.038-4.936 |

* eGFR, estimated glomerular filtration (CKD-EPI equation for creatinine, ml/min/1.73m²); uDKK3, urinary Dickkopf-3.

** HR per doubling increase in uDKK3.

**Table S9.** Univariate and multivariate Cox regression analysis for mortality event for per doubling of uDKK3 adjusted for age, log proteinuria, eGFR and interstitial fibrosis

|  | HR | CI (95%) |
| --- | --- | --- |
| **Univariate analysis** |  |  |
| uDKK3 (per doubling) | 3.647 | 1.261-10.548 |
| **Multivariate analysis** |  |  |
| Age (years) | 1.031 | 0.995-1.069 |
| Log Proteinuria (mg) | 1.577 | 0.885-2.808 |
| eGFR (ml/min/1.73m2) | 0.973 | 0.945-1.003 |
| Interstitial fibrosis | 1.008 | 0.967-1.050 |
| uDKK3 (per doubling) | 1.695 | 0.461-6.231 |

* eGFR, estimated glomerular filtration (CKD-EPI equation for creatinine, ml/min/1.73m²); uDKK3, urinary Dickkopf-3.

** HR per doubling increase in uDKK3.

**Figure S1.** ROC curves for predicting interstitial fibrosis >25% in the biopsy based on levels of tubular biomarkers.


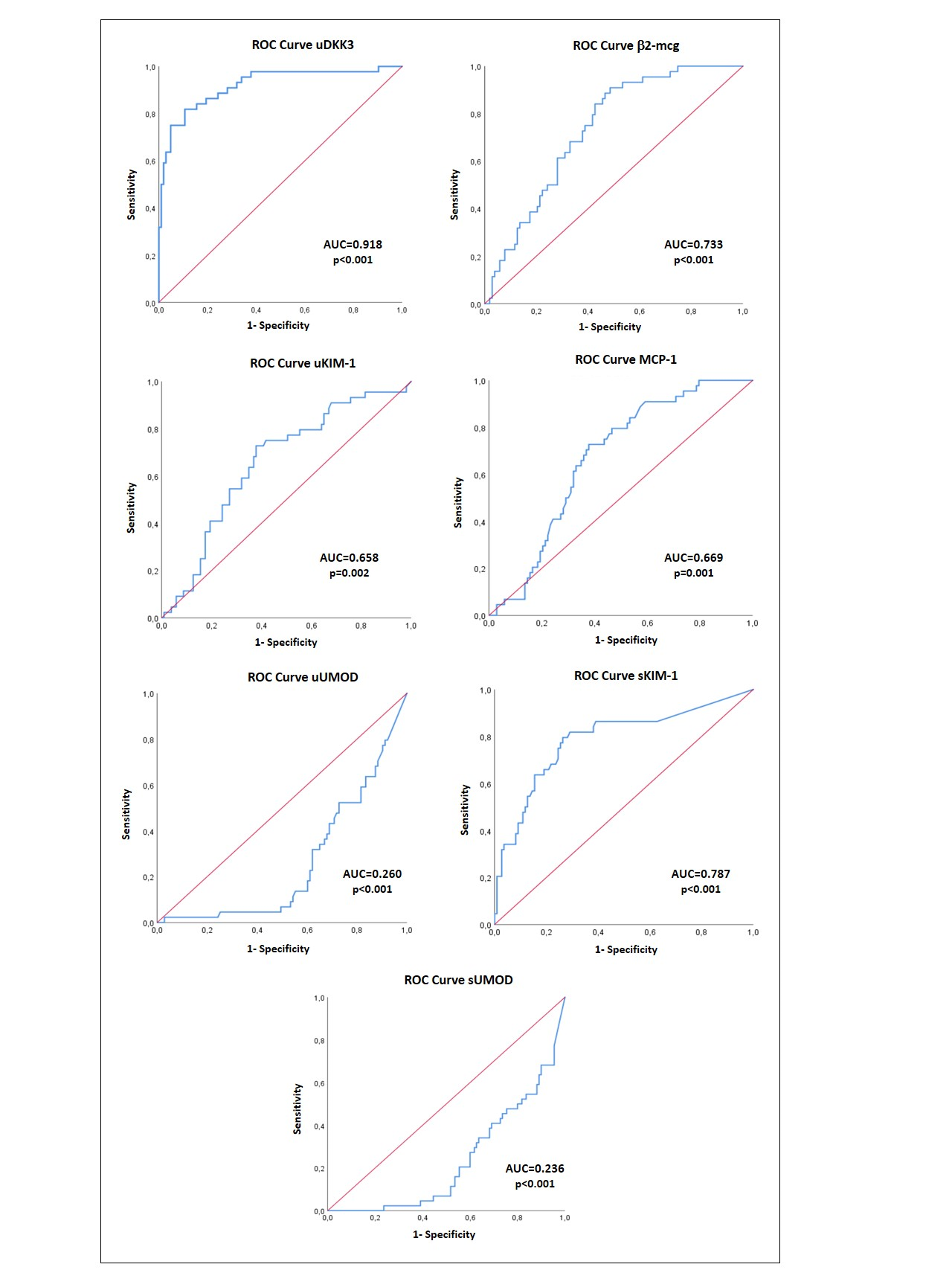


*β2-mcg, beta 2 microglobulin; uKIM-1, urine Kidney Injury Molecule-1 urine; MCP-1, Monocyte Chemoatractant Protein 1; uDKK3, urinary Dickkopf-3; uUMOD, urinary uromodulin; sKIM-1, serum Kidney Injury Molecule-1; sUMOD, serum uromodulina; AUC, area under the curve.

**Figure S2.** Box-plot distribution of median tubular markers according to the degree of cortical interstitial inflammation in the biopsy.


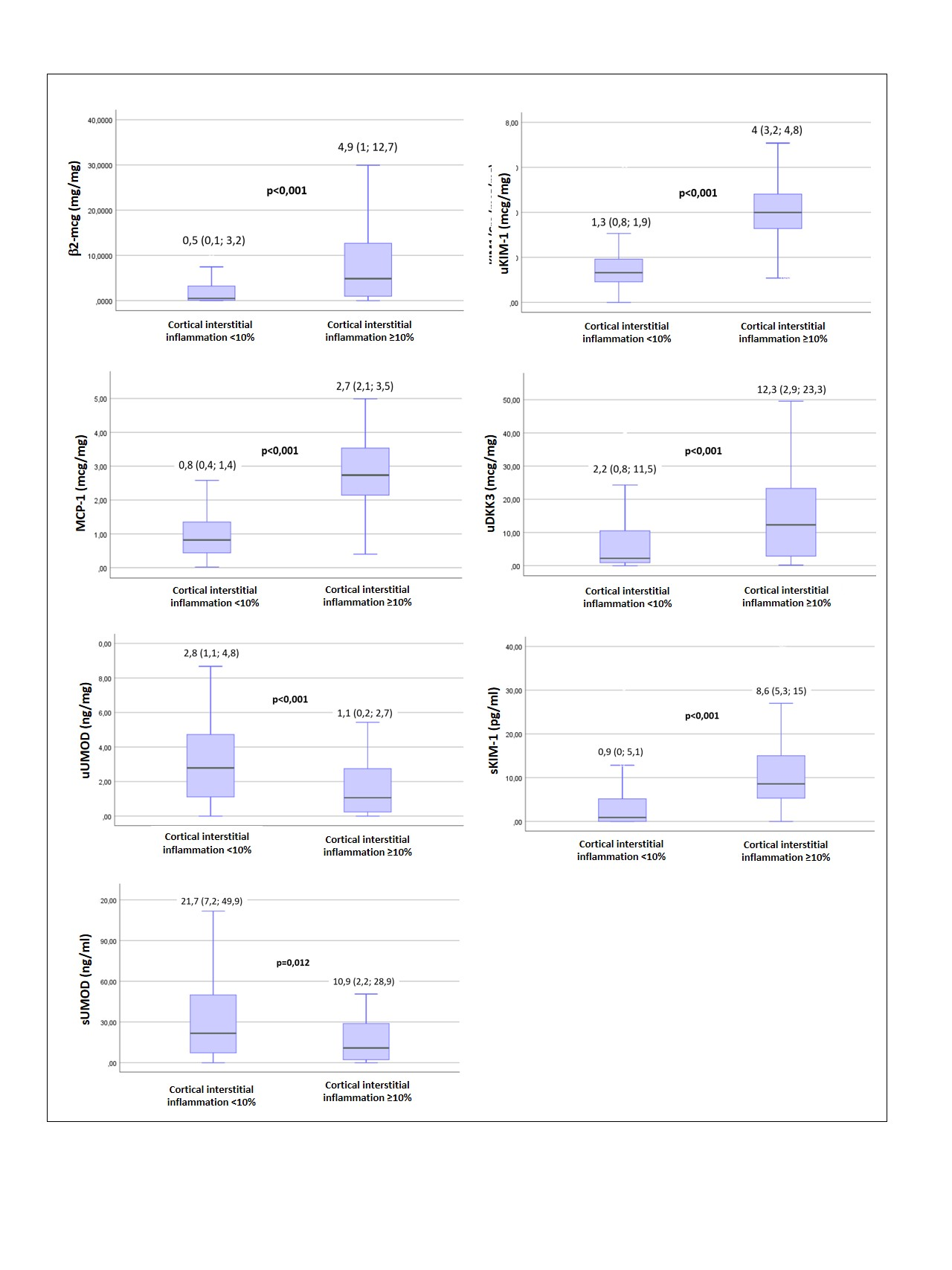


* β2-mcg, beta 2 microglobulin; uKIM-1, urine Kidney Injury Molecule-1 urine; MCP-1, Monocyte Chemoatractant Protein 1; uDKK3, urinary Dickkopf-3; uUMOD, urinary uromodulin; sKIM-1, serum Kidney Injury Molecule-1; sUMOD, serum uromodulin.

**Mann-Whitney U test for independent samples, significant if p <0.05.
